# Risk factors for anti‐drug antibody formation to infliximab: Secondary analyses of a randomised controlled trial

**DOI:** 10.1111/joim.13495

**Published:** 2022-04-26

**Authors:** Marthe Kirkesæther Brun, Guro Løvik Goll, Kristin Kaasen Jørgensen, Joseph Sexton, Johanna Elin Gehin, Øystein Sandanger, Inge Christoffer Olsen, Rolf Anton Klaasen, David John Warren, Cato Mørk, Tore K. Kvien, Jørgen Jahnsen, Nils Bolstad, Espen A. Haavardsholm, Silje Watterdal Syversen

**Affiliations:** ^1^ Division of Rheumatology and Research Diakonhjemmet Hospital Oslo Norway; ^2^ Institute of Clinical Medicine University of Oslo Oslo Norway; ^3^ Department of Gastroenterology Akershus University Hospital Lørenskog Norway; ^4^ Department of Medical Biochemistry Oslo University Hospital Oslo Norway; ^5^ Section of Dermatology Oslo University Hospital Oslo Norway; ^6^ Department of Research Support for Clinical Trials Oslo University Hospital Oslo Norway; ^7^ Akershus Dermatology Center Lørenskog Norway

**Keywords:** autoimmune disease, immunosuppressive treatment

## Abstract

**Background:**

Anti‐drug antibodies (ADAb) frequently form early in the treatment course of infliximab and other tumour necrosis factor (TNF) inhibitors, leading to treatment failure and adverse events.

**Objective:**

To identify risk factors for ADAb in the early phase of infliximab treatment.

**Methods:**

Patients (*n* = 410) with immune‐mediated inflammatory diseases who initiated infliximab treatment were included in the 38‐week Norwegian Drug Monitoring Trial (NOR‐DRUM) A and randomised 1:1 to therapeutic drug monitoring (TDM) or standard therapy. Serum levels of infliximab and ADAb were measured at each infusion. Possible risk factors for ADAb formation were assessed using logistic regression, adjusting for potential confounders.

**Results:**

ADAb were detected in 78 (19%) patients. A diagnosis of rheumatoid arthritis (RA) (odds ratio [OR], 1.9 [95% confidence interval [CI] 1.0–3.6]) and lifetime smoking (OR, 2.0 [CI 1.1–3.6]) were baseline risk factors, while baseline use of concomitant immunosuppressors (OR, 0.4 [CI 0.2–0.8]) and a diagnosis of spondyloarthritis (SpA) (OR, 0.4 [CI 0.2–0.8]) reduced the risk of ADAb. Higher disease activity during follow‐up (OR, 1.1 [CI 1.0–1.1]) and “drug holidays” of more than 11 weeks (OR, 4.1 [CI 1.2–13.8]) increased the risk of ADAb, whereas higher infliximab doses (OR, 0.1 [CI 0.0–0.3) and higher serum infliximab concentrations (OR, 0.7 [CI 0.6–0.8]) reduced the risk of immunogenicity.

**Conclusion:**

Several risk factors for ADAb formation during early‐phase infliximab treatment were identified. This knowledge provides a basis for treatment strategies to mitigate the formation of ADAb and identify patients in whom these measures are of particular importance.

## Graphical abstract

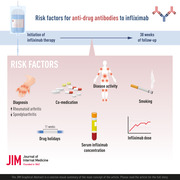

## Introduction

Tumour necrosis factor α inhibitors (TNFi), including infliximab, have revolutionised the treatment of prevalent chronic immune‐mediated inflammatory diseases such as rheumatoid arthritis (RA), psoriatic arthritis (PsA), spondyloarthritis (SpA), ulcerative colitis (UC), Crohn's disease (CD), and psoriasis (Ps). However, lack or loss of response to treatment, leading to increased disability, reduced quality of life, and potential irreversible organ damage, is seen in up to 55% of patients during the first 6 months of treatment [1, 2, 3, 4].

Immunogenicity is a major concern associated with TNFi use. The formation of neutralising anti‐drug antibodies (ADAb) which inactivate the therapeutic agent is a leading cause of TNFi treatment failure [5, 6, 7, 8, 9]. ADAb formation is also related to safety issues and frequently leads to infusion reactions in patients on infliximab therapy [5, 10, 11]. Immunogenicity is of particular concern early in the treatment course [12]. Infliximab, a murine‐human chimeric antibody, exhibits a greater immunogenic potential than other TNFi, and ADAb eventually develop in approximately one out of four patients [7].

Data to help identify patients at risk of developing ADAb are scarce. The use of concomitant immunosuppressive medications, such as methotrexate and azathioprine, has been suggested to prevent ADAb formation [7, 13] whereas smoking and prior use of TNFi may increase the risk of ADAb formation [14, 15]. Low serum infliximab concentrations have been proposed to trigger immunogenicity [16], but a cut‐off associated with this increased risk has not yet been identified. Studies examining risk factors and mitigation strategies across disease groups are lacking.

The Norwegian Drug Monitoring Trial (NOR‐DRUM) A randomised clinical trial assessed the effectiveness of proactive therapeutic drug monitoring (TDM) during induction of infliximab therapy and did not find TDM to be superior to standard infliximab therapy in terms of achieving clinical remission in the overall population [17]. However, the subgroup of patients who subsequently developed ADAb benefited from TDM during induction therapy, suggesting that TDM might be a beneficial treatment strategy in patients at increased risk of immunogenicity [18].

Identifying patients at risk of ADAb formation is clinically important for the timely detection of ADAb to prevent treatment failure and immunogenicity‐related side effects. Increased knowledge of risk factors will facilitate the choice of treatment strategies to mitigate the formation of ADAb.

The aim of this sub‐study of the NOR‐DRUM A trial [17] was to identify patient‐ and treatment‐related risk factors for ADAb formation, including the relationship between infliximab serum concentrations and ADAb during the early phases of infliximab therapy.

## Methods

### Population and study design

The randomised controlled NOR‐DRUM A trial assessed the effectiveness of TDM compared to standard infliximab therapy [17]. Adult patients (*n* = 411) with an immune‐mediated inflammatory disease (84 (20%) RA, 45 (11%) PsA, 119 (29%) SpA, 83 (20%) UC, 58 (14%) CD, and 22 (5%) Ps) initiating infliximab therapy were enrolled in the 38‐week trial. The patients were randomised 1:1 to TDM or standard infliximab therapy. The starting dose of infliximab was according to the summary of product characteristics: 3 mg/kg for patients with RA and 5 mg/kg for patients with PsA, SpA, UC, CD, and Ps. Study visits were scheduled at each infusion: weeks 0, 2, and 6 and every 6–10th week thereafter. In the TDM arm, adjustments in infliximab dose and infusion intervals were performed in accordance with an algorithm that included the serum concentrations of infliximab and ADAb. This algorithm advised increasing the infliximab dose if ADAb were detected at concentrations ≤50 µg/L, whereas switching to another therapy was advised if the ADAb concentration was >50 µg/L (Appendix Table 1 in the Supporting Information). The 50 µg/L cut‐off was established after considering prior observational data using the same assay, demonstrating that ADAb above this level were rarely transient [17]. In the standard therapy arm, infliximab administration was based only on clinical judgment. The concentrations of infliximab and ADAb were assessed in all patients at each infusion, but the results for patients in the standard therapy arm were not made available to study personnel. Treatment with concomitant immunosuppressive medication was at the discretion of the treating physician for both groups. The primary endpoint was remission at week 30. Further details regarding study design and patient disposition have been described elsewhere [17, 19]. The study (ClinicalTrials.gov number NCT‐03074656) was conducted in accordance with the Declaration of Helsinki and approved by the Norwegian Regional Committees for Medical and Health Research Ethics. Written informed consent was obtained from all the patients.

### Study assessments and outcome

Demographic data (sex, age, tobacco and snuff use, and coffee consumption), diagnosis, and use of immunosuppressive comedication were recorded at baseline. Infliximab treatment regimen (starting dose, drug doses during follow‐up, infusion intervals, and treatment intensifications), serum concentrations of infliximab and ADAb, and changes in co‐medication were recorded at each study visit. Disease activity parameters (disease‐specific composite scores, patient global assessment, physician global assessment, C‐reactive protein (CRP), erythrocyte sedimentation rate (ESR), and faecal calprotectin levels [in UC and CD]) were recorded at baseline and during follow‐up [17]. The main disease activity measures for the six diseases were the Disease Activity Score in 28 joints (DAS28) for RA and PsA, the Ankylosing Spondylitis Disease Activity Score (ASDAS) for SpA, the Partial Mayo Score (PMS) for UC, the Harvey‐Bradshaw index (HBI) for CD, and the Psoriasis Area and Severity Index (PASI) for Ps [17]. Blood samples were collected within 1 week before each infusion (trough) for all participants. Serum samples for infliximab and ADAb measurements were analysed at a central laboratory (Department of Medical Biochemistry, Oslo University Hospital, Radiumhospitalet) using in‐house assays automated on the AutoDELFIA (PerkinElmer, Waltham, MA, USA) immunoassay platform [20]. Analyses of ADAb were performed for all samples with serum drug concentrations <5 mg/L.

The ADAb assay is a drug‐sensitive inhibition assay that only detects neutralising ADAb, using biotinylated recombinant human TNF on the solid phase and europium‐labelled infliximab F(ab’)_2_ as tracer. The assay is calibrated against recombinant anti‐infliximab antibody (Bio‐Rad, clone AbD20436 hIgG1). Appendix Table 2 in the Supporting Information provides a more detailed description of this assay. ADAb were defined as positive if its concentration was ≥15 µg/L.

### Statistical analysis

The outcome of this study was ADAb formation in patients on infliximab therapy. In patients who discontinued infliximab treatment, ADAb had to be detected within 12 weeks after the last infusion for the patient to be registered as ADAb‐positive. Logistic regression analyses were performed to assess baseline risk factors, as well as treatment‐ and disease‐related risk factors during follow‐up. For the follow‐up variables, data up to, but not including, the first date of detection of ADAb in ADAb‐positive patients were included. All variables were assessed in univariate analyses, and variables with a *p* value <0.25 were further examined in multivariate analyses adjusting for age, sex, diagnosis, and disease activity. To adjust for disease activity, despite disease activity scores on different scales, a standardised variable was computed using the *z*‐score for each patient (subtracting the mean of the variable from the raw values and dividing by the SD of the variable). Sensitivity analyses with adjustment for CRP and without adjustment for disease activity, respectively, were also performed. Multiplicity adjustment was done using the Benjamini and Hochberg procedure with the false discovery rate set to 5% [21]. The subgroup analyses were adjusted for age, sex, and disease activity. Patients with missing independent variable data were not included in the analyses of the relevant variables. The cumulative hazard functions for each diagnosis were examined using the Nelson‐Aalen estimator [22].

The occurrence of ADAb according to infliximab concentration was assessed to explore the concentration/risk relationship. In patients with ADAb formation, infliximab concentrations up to but not including the first time point with ADAb detection were included in the analyses. As infliximab treatment follows an induction regimen, the concentration/risk relationship was assessed separately for drug concentrations before infusions 2 and 3 (induction phase) and after infusion 3 (maintenance phase). Drug concentrations in the maintenance phase were summarised into a mean value for each patient. To explore the association between mean infliximab concentration and the probability of ADAb development, we grouped patients according to concentration deciles and estimated the probability of developing ADAb separately in each group. To determine a cut‐off for infliximab concentrations that could best stratify patients with and without ADAb, receiver operating characteristic curve (ROC) analyses were performed. Statistical analyses were performed using STATA v.16 (StataCorp). *p* values <0.05 were considered statistically significant.

### Role of the funding source

The funder of the study (The Norwegian Regional Health Authorities) had no role in the study design or conduct; data collection, management, analysis, or interpretation; manuscript preparation, review, or approval; nor in the decision to submit the manuscript for publication.

## Results

### Description of the study cohort

Of the 411 patients in the NOR‐DRUM A trial, 410 underwent at least one serum infliximab concentration assessment and were included in the present analyses. Table 1 presents the demographic and disease‐ and treatment‐related characteristics stratified by ADAb status. In total, 106 (26%) had used prior biologic therapy and 226 (55%) received concomitant immunosuppressive therapy at baseline, mainly methotrexate and thiopurines (Table 1). Additionally, 22 patients started methotrexate comedication and 24 patients started thiopurine comedication during follow‐up. Systemic glucocorticoid treatment was administered to 131 (32%) patients at some point during the study (73 (18%) at baseline), 70 of which used high‐dose prednisolone (≥15 mg/kg). The median infliximab dose during the maintenance phase was 5.0 mg/kg (interquartile range [IQR] 4.7–5.2). As shown in Appendix Table 3 in the Supporting Information, patients with RA and UC had the lowest and highest weekly drug doses, respectively, in the maintenance phase. Overall, median infliximab serum concentrations were 29.5 (IQR 21.0–36.9), 24.8 (IQR 16.7–32.1), and 7.0 (IQR 4.1–11.3) mg/L at infusions 2 and 3 and during the maintenance phase, respectively. The lowest infliximab concentrations during the maintenance phase were observed in patients with RA, and the highest in patients with UC (Appendix Table 3 in the Supporting Information). Infliximab was discontinued in 113 (28%) patients, of whom 44 were ADAb‐positive.

**Table 1:** Demographic, disease, and treatment‐related characteristics, stratification according to ADAb status

	All patients	Patients without ADAb formation	Patients with ADAb formation
	(*n* = 410)	(*n* = 332)	(*n* = 78)
**Demographics**			
Median age (IQR), years	44 (32–56)	44 (30–55)	47 (37–58)
Female sex, *n* (%)	209 (51)	166 (50)	43 (55)
Median disease duration (IQR), years	3.6 (0.8–12.9)	3.6 (0.8–13.4)	4.3 (0.8–12.5)
**Diagnoses** ^a^			
Rheumatoid arthritis, *n* (%)	84 (21)	59 (70)^a^	25 (30)^a^
Psoriatic arthritis, *n* (%)	44 (10)	33 (75)^a^	11 (25)^a^
Spondyloarthritis, *n* (%)	119 (29)	106 (89)^a^	13 (11) ^a^
Ulcerative colitis, *n* (%)	83 (20)	68 (82)^a^	15 (18)^a^
Crohn's disease, *n* (%)	58 (14)	49 (85)^a^	9 (16)^a^
Psoriasis, *n* (%)	22 (5)	17 (77)^a^	5 (23)^a^
**Therapy**			
Prior use of biologic therapy, *n* (%)	106 (26)	90 (27)	16 (21)
Prior use of one or more TNF inhibitors^b^, *n* (%)	94 (23)	80 (24)	14 (18)
Prior use of other biologics^c^, *n* (%)	17 (4)	15 (5)	2 (3)
**Concomitant immunosuppressive therapy at baseline** ^d^, *n* (%)	226 (55)	185 (56)	41 (53)
Concomitant use of methotrexate, *n* (%)	154 (38)	123 (37)	31 (40)
Median dose (IQR), mg/week	20 (15–23)^e^	20 (15–20)	20 (15–25)
Concomitant use of thiopurines, *n* (%)	55 (13)	41 (15)	4 (5)
Median dose (IQR), mg/day	100 (50–100)^f^	100 (50–100)	75 (50–250)
Concomitant use of glucocorticoids, *n* (%)	73 (18)	59 (18)	14 (18)
Prednisolone ≥15mg/day, *n* (%)	30 (7)	28 (8)	2 (2)
**General baseline characteristics**			
Median ESR^h^ (IQR), mm/h	13.0 (6.0–25.0)	13.0 (6.0–25.0)	16.0 (9.0–24.0)
Median CRP (IQR), mg/L	5.0 (1.0–14.0)	5.0 (1.0–15.0)	4.5 (2.0–12.0)
Mean patient's global assessment of disease activity^g^, ^h^ (SD), mm	58.3 (22.9)	59.3 (22.6)	53.9 (23.9)
Mean physician's global assessment of disease activity^g^, ^h^ (SD), mm	46.5 (21.4)	47.1 (21.1)	44.1 (22.7)
Lifetime smoking, *n* (%)	233 (57)	177 (53)	56 (72)
Median infliximab starting dose (IQR), mg/kg	5.0 (4.7–5.2)	5.0 (4.7–5.3)	4.9 (3.1–5.2)
**Disease specific baseline characteristics**			
**Rheumatoid arthritis**			
Anti‐citrullinated protein antibody positive, *n* (%)	57 (68)	42 (71)	15 (60)
Rheumatoid factor positive, n (%)	55 (66)	40 (69)	15 (60)
Mean DAS28 (SD)	4.5 (1.2)	4.4 (1.2)	4.6 (1.2)
Concomitant immunosuppressive therapy, *n* (%)	78 (92)	55 (93)	23 (92)
**Psoriatic arthritis**			
Mean DAS28 (SD)	4.5 (1.2)	4.6 (1.2)	4.2 (1.0)
Concomitant immunosuppressive therapy, *n* (%)	35 (80)	26 (79)	9 (82)
**Spondyloarthritis**			
Mean ASDAS (SD)	3.1 (1.0)	3.1 (1.0)	3.2 (0.8)
Concomitant immunosuppressive therapy, *n* (%)	27 (23)	27 (26)	0 (0)
**Ulcerative colitis**			
Median PMS (IQR)	6 (4–7)	6 (5–7)	6 (2–7)
Concomitant immunosuppressive therapy, *n* (%)	33 (40)	30 (44)	3 (20)
**Crohn's disease**			
Median HBI (IQR)	8 (6–10)	8 (6–10)	7 (6–9)
Concomitant immunosuppressive therapy, *n* (%)	37 (64)	34 (70)	3 (33)
**Psoriasis**			
Mean PASI (SD)	9.4 (7.4–10.8)	10.2 (7.6–11.1)	8.6 (6.1–9.4)
Concomitant immunosuppressive therapy, *n* (%)	16 (73)	13 (77)	3 (60)

Data are *n* (%), mean (SD) or median (IQR).

ADAb, anti‐drug antibody; TNF, tumour necrosis factor; ESR, erythrocyte sedimentation rate; CRP, C‐reactive protein; DAS28, Disease Activity Score in 28 Joints; HLA, human leukocyte antigen; ASDAS, Ankylosing Spondylitis Disease Activity Score; HBI, Harvey‐Bradshaw Index; PMS, Partial Mayo Score; PASI, Psoriasis Area and Severity Index.

^a^ Percentage is the proportion with/without ADAb formation within the disease group.

^b^ Prior TNFi include: Etanercept, adalimumab, certolizumab pegol, golimumab, and infliximab.

^c^ Other biologics include: abatacept, rituximab, secukinumab, tocilizumab, ustekinumab, and vedolizumab.

^d^ Concomitant immunosuppressive medication include: methotrexate, azathioprine, leflunomide (*n* = 5), and sulfasalazine (*n* = 12).

^e^ Methotrexate doses: 55% received ≥20 mg/week, 24% received 15–20 mg/week, and 21% received <15 mg/week.

^f^ Thiopurine doses: 45% received 100 mg/day and 38% received 50 mg/day.

^g^ Global assessment of disease activity range, 0 to 100 on a visual analogue scale, with 0 indicating no disease activity and 100 indicating the highest possible disease activity.

^h^ Data were missing for some patients.

### Anti‐drug antibody formation

**Fig. 1:**
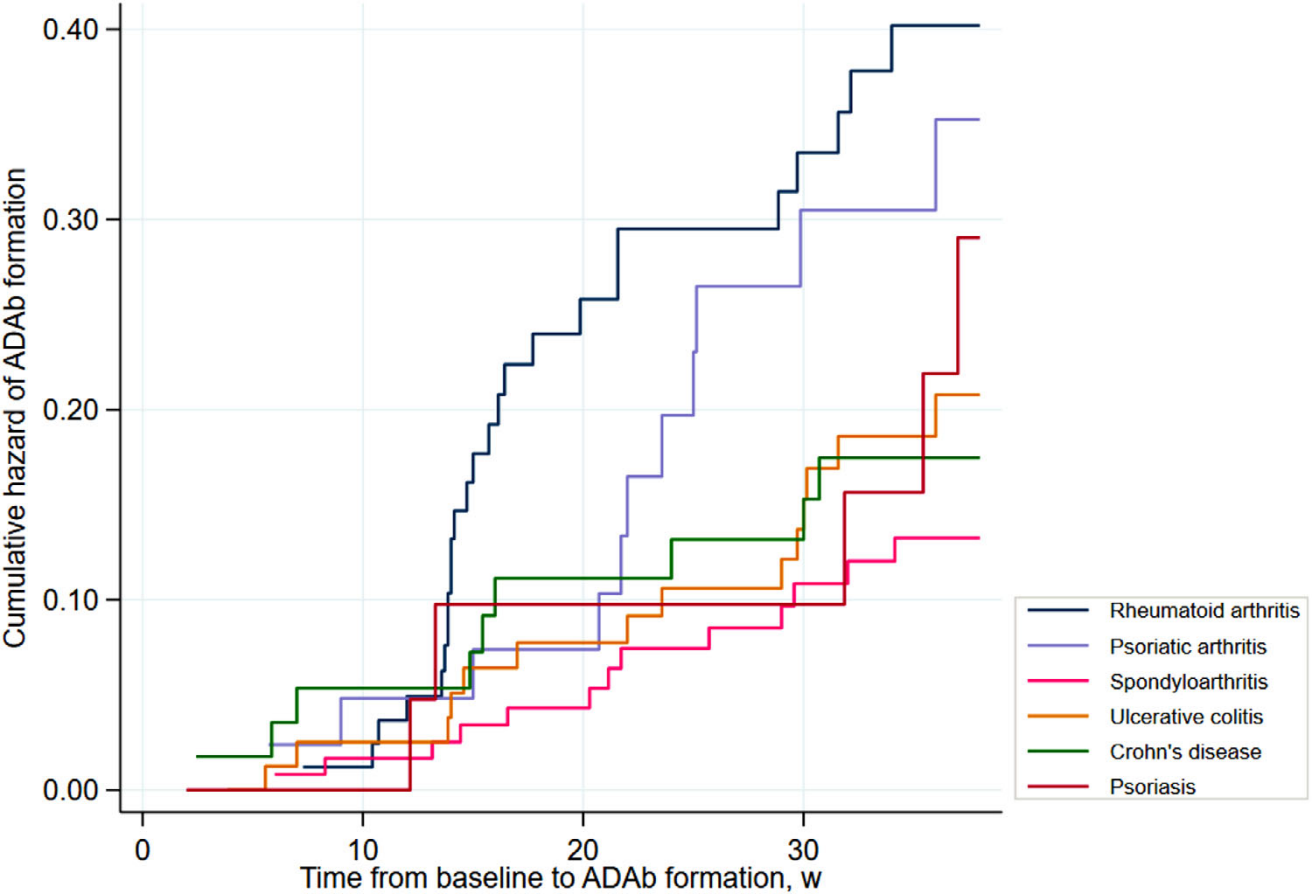
Nelson‐Aalen plot showing the cumulative hazard of ADAb formation for each diagnosis. Each step indicates a new event. The slope of the curve indicates the hazard rate. ADAb, anti‐drug antibodies; w, weeks.

ADAb formation while on infliximab therapy was detected in 78 (19%) patients (RA 25/84 (30%), PsA 11/44 (25%), SpA 13/119 (11%), UC 15/83 (18%), CD 9/58 (16%), Ps 5/22 (23%)) (Table 1). Additionally, ADAb were detected in 26 patients after the discontinuation of infliximab. The mean time from baseline to ADAb detection was 19.7 weeks (95% confidence interval [CI], 17.6–21.8). Figure 1 shows the cumulative hazard function for each diagnosis. Nine patients had transient ADAb (concentration range 16–89 µg/L). Detailed information about these patients is provided in Appendix Table 4 in the Supporting Information.

In the standard therapy arm reflecting standard care, 10/36 (27.8%) of patients with ADAb versus 72/168 (42.9%) of patients without ADAb, achieved remission. Additionally, a large proportion of patients with ADAb discontinued infliximab (14/36 (39%) versus 33/135 (16.6%)) or had an infusion reaction (12/36 (33.3%) versus 4/168 (2.4%)), compared to patients without ADAb.

### Risk factors for ADAb formation

Baseline risk factors of ADAb (Table 2) included a diagnosis of RA (odds ratio [OR], 1.9 [CI 1.0–3.6]) and lifetime smoking (OR, 2.0 [CI 1.1–3.6]), whereas a diagnosis of SpA (OR, 0.4 [CI 0.2–0.8]) and concomitant immunosuppression (OR, 0.4 [CI 0.2–0.8]) were shown to reduce the risk of ADAb (Table 2).

**Table 2:** Baseline risk factors for anti‐drug antibody formation

	Anti‐drug antibody formation, *n*=78/410			
	Univariate analysis		Adjusted analysis	
	OR [95% CI]	*p* value	OR [95% CI]	*p* value
Age	1.02 [1.00–1.04]	0.03	1.01 [0.99–1.03]	0.17
Diagnosis of rheumatoid arthritis (*n*=84/410)	2.18 [1.26–3.79]	<0.01	1.93 [1.04–3.60]	0.04
Diagnosis of spondyloarthritis (*n*=119/410)	0.43 [0.23–0.81]	<0.01	0.41 [0.21–0.79]	<0.01
Prior use of ≥1 TNF inhibitor(s) (*n*=94/410)	0.69 [0.37–1.29]	0.25	0.61 [0.31–1.20]	0.16
Concomitant immunosuppressive therapy (*n*=226/410)	0.88 [0.54–1.44]	0.61^a^	0.40 [0.21–0.76]	<0.01
Concomitant use of prednisolone ≥15 mg/day (*n*=30/410)	0.29 [0.07–1.23]	0.09	0.26 [0.06–1.16]	0.08
Patient's global assessment of disease activity, VAS (0–100 mm) (*n*=408^b^)	0.99 [0.98–1.00]	0.06	0.99 [0.98–1.00]	0.20
Infliximab starting dose (mg/kg) (*n*=410)	0.75 [0.60–0.93]	0.01	0.84 [0.60–1.17]	0.29
Lifetime smoking (*n*=233/410)	2.23 [1.30–3.82]	<0.01	1.98 [1.11–3.55]	0.02

Results are presented as odds ratios (OR) with 95% confidence intervals (CI). The adjusted analysis was corrected for age, gender, diagnosis, and a standardised disease activity score. All variables with a *p* value <0.25 in the univariate analysis were included in the adjusted analyses. Variables tested, but not associated with ADAb (*p* values >0.25 in univariate analysis) included: gender, BMI, coffee drinking, snuff consumption, disease duration, randomization (TDM/standard care), prior non‐TNFi treatment, physician's global assessment of disease activity and methotrexate and thiopurine dosing.

TNF, tumour necrosis factor.

^a^ Included in table despite an unadjusted *p* value>0.25 because this variable was significant in adjusted analysis.

^b^ Data were missing in some patients.

During follow‐up (Table 3), higher disease activity assessed by mean ESR (OR, 1.1 [CI 1.0‐1.1]) and mean CRP level (OR, 1.1 [CI 1.0–1.1]) and “drug holidays” of more than 11 weeks (OR, 4.1 [CI 1.2–13.8]) increased the risk of ADAb, whereas higher infliximab doses (OR, 0.1 [CI 0.0–0.3]) and higher serum infliximab concentrations (OR, 0.7 [CI 0.6–0.8]) were associated with reduced risk of immunogenicity.

**Table 3:** Patient and treatment related risk factors for anti‐drug antibody formation during follow‐up

	Anti‐drug antibody formation, *n*=77/406^a^			
	Univariate analysis		Adjusted analysis	
	OR [95% CI]	*p* value	OR [95% CI]	*p* value
Concomitant use of systemic glucocorticoids after baseline (*n*=58/406)	5.19 [2.86–9.43]	<0.001	5.53 [2.90–10.55]	<0.001
Infliximab dose/week infusion 1 and 2 (mg/kg/week) (*n*=390^b^)	0.43 [0.23–0.79]	<0.01	0.59 [0.26–1.32]	0.20
Infliximab dose/week infusion 3 and onwards (mg/kg/week) (*n*=355^c^)	0.11 [0.04–0.35]	<0.001	0.08 [0.02–0.31]	<0.001
One or more infliximab dose increment(s) (*n*=148/406)	0.51 [0.29–0.89]	0.02	0.43 [0.24–0.78]	<0.01
More than 11 weeks between infusions (*n*=13/406)	3.89 [1.27–11.92]	0.02	4.12 [1.23–13.75]	0.02
Mean CRP level (*n*=406)	1.05 [1.01–1.08]	<0.01	1.05[1.02–1.09]	<0.01
Mean ESR level (*n*=406)	1.05 [1.03–1.07]	<0.001	1.05 [1.02–1.08]	<0.001
Infliximab concentration before infusion 2 (*n*=398^d^)	0.95 [0.93–0.97]	<0.001	0.96 [0.93–0.98]	<0.01
Infliximab concentration before infusion 3 (*n*=389^e^)	0.91 [0.88–0.94]	<0.001	0.91 [0.89–0.94]	<0.001
Mean infliximab concentration after infusion 3 (*n*=326^f^)	0.74 [0.65–0.83]	<0.001	0.73 [0.64–0.84]	<0.001

Results are presented as odds ratios (OR) with 95% confidence intervals (CI). The adjusted analyses are corrected for age, gender, diagnosis, and a standardised disease activity score. All variables with a *p* value <0.25 in univariate analysis included. Variables tested, but not associated with ADAb (*p* value >0.25 in univariate analysis) include mean patient's global assessment of disease activity, mean physician's global assessment of disease activity, starting or terminating concomitant immunosuppressive medication, and having one or more infection(s).

ESR, erythrocyte sedimentation rate; CRP, C‐reactive protein.

^a^ Four patients did not have assessments after baseline.

^b^ Some patients did not have more than one infusion.

^c^ Some patients did not have three or more infusions.

^d^ Some patients did not have more than one infusion or did not have a serum infliximab assessment before infusion 2.

^e^ Some patients did not have more than two infusions or did not have a serum infliximab assessment before infusion 3.

^f^ Some patients did not have more than three infusions or did not have serum infliximab assessments after infusion

These findings were consistent in the univariate and multivariate analyses, except for the use of concomitant immunosuppressive therapy, which was not significantly associated with ADAb formation in the univariate analysis (Table 2). Variables with *p* values <0.25 in the univariate analyses are shown in Tables 2 and 3, and variables with *p* values >0.25 are listed in the table legends. Sensitivity analyses with adjustment for CRP level and without adjustment for disease activity yielded similar results (Appendix Table 5 in the Supporting Information).

A total of 37 potential risk factors were assessed in the univariate analyses (Tables 2 and 3), of which 15 had univariate associations with *p* values <0.05. Twelve of the 15 remained significant after controlling for multiple testing, using a false discovery rate of 5%. The three that did not stand up to multiplicity adjustment in the univariate analyses were having more than 11 weeks between infusions, having one or more infliximab dose increments, and age (Appendix Table 6 in the Supporting Information).

### Risk factors for ADAb formation in disease subgroups

Subgroup analyses in patients with peripheral arthritis (RA and PsA, *n* = 128), SpA (*n* = 119), and inflammatory bowel disease (UC and CD, *n* = 141) were performed to assess disease‐specific risk factors for ADAb formation (Appendix Table 7 in the Supporting Information). The number of patients with Ps (*n* = 22) was too low to allow separate analyses in this subgroup. Higher infliximab concentrations during the induction and maintenance phases reduced the risk of ADAb formation in all subgroups. In the peripheral arthritis subgroup, we found lifetime smoking (OR, 3.0 [CI 1.1–8.4]) to be a risk factor for ADAb formation, in addition to systemic glucocorticoid therapy after baseline (OR, 3.4 [CI 1.2–9.5]), higher mean DAS28 score (OR, 1.5 [CI 1.1–2.3]), and higher mean ESR (OR, 1.1 [CI 1.0–1.1]) and mean CRP levels (OR, 1.1 [CI 1.0–1.2]). In the SpA subgroup, higher mean ESR (OR, 1.1 [CI 1.0–1.1]) and mean CRP levels (OR, 1.1 [CI 1.0–1.1]) were risk factors for immunogenicity. None of the 27 patients with SpA and comedication at baseline (25 methotrexate and 2 sulfasalazine) had ADAb formation. In the inflammatory bowel disease subgroup, higher age (OR, 1.0 [CI 1.0–1.1]), systemic glucocorticoid therapy after baseline (OR, 9.5 [CI 3.2–28.2]), higher mean ESR (OR, 1.1 [CI 1.0–1.1]), and higher mean white blood cell count (OR, 1.3 [CI 1.0–1.8]) were risk factors for ADAb formation. Higher infliximab doses in the maintenance phase (OR, 0.1 [CI 0.0–0.4]), having one or more infliximab dose increments (OR, 0.3 [CI 0.1–0.9]), and concomitant use of thiopurines (OR, 0.3 [CI 0.1–0.9]) reduced the risk of immunogenicity. The nonsignificant disease‐specific factors tested are shown in Appendix Table 7 in the Supporting Information.

### Relationship between infliximab concentration and ADAb

**Fig. 2:**
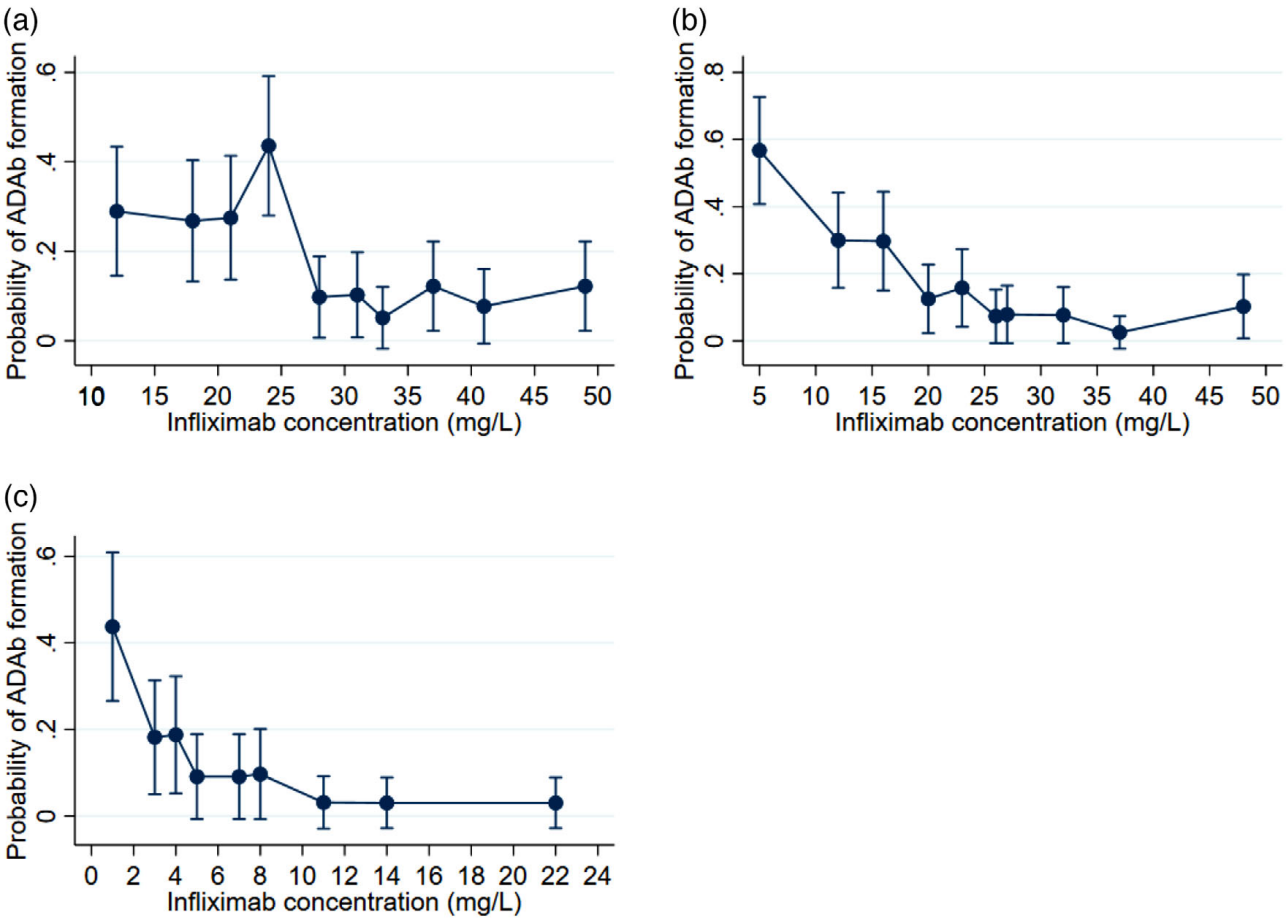
Concentration–risk curves. Predicted probability of later ADAb formation with CI for infliximab trough serum concentrations before infusion 2 (a), before infusion 3 (b) and in the maintenance phase (c). Patients were sorted by infliximab concentrations (low to high) and divided into equally sized groups (n = 10). Mean values were calculated for each group. Predictive values were obtained from univariate regression analyses. ADAb, anti‐drug antibodies; CI, 95% confidence interval.

**Fig. 3:**
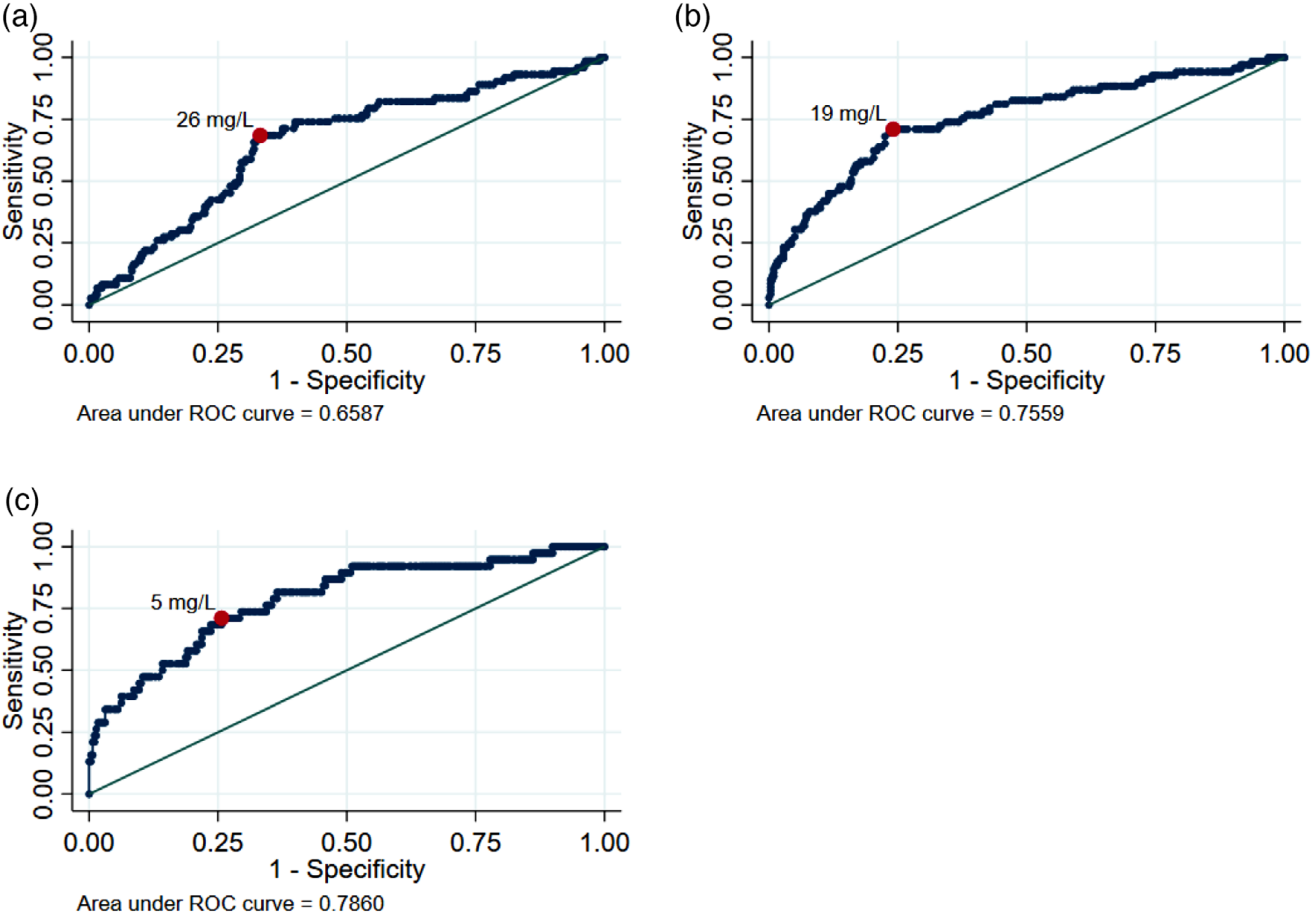
Receiver operating characteristics curves with AUC to identify cut‐offs for infliximab trough serum concentrations for best discrimination of patients with and without later ADAb formation. Predictive values were obtained from univariate regression analyses. The optimal cut‐off point was defined by the point closest to 0.1 (i.e., 100% sensitivity and specificity). (a). Infliximab concentrations before infusion 2. AUC is 0.66 (CI 0.61–0.71) with optimal cut‐off at 26 mg/L with a sensitivity of 68.5% (CI 57.1–78.0) and specificity of 66.8% (CI 61.5–71.7). (b). Infliximab concentrations before infusion 3. AUC is 0.76 (CI 0.71–0.80) with optimal cut‐off at 19 mg/L with a sensitivity of 71.0% (CI 59.4–80.4) and a specificity of 75.9% (CI 71.0–80.3). (c). Infliximab concentrations in the maintenance phase (calculated mean for each patient). AUC is 0.79 (CI 0.74–0.83) with optimal cut‐off at 5 mg/L with a sensitivity of 71.1% (CI 55.2–83.0) and specificity of 74.3% (CI 69.0–79.0). AUC, area under the curve; ADAb, anti‐drug antibody; CI, 95% confidence interval.

The relationship between infliximab concentration and risk of ADAb formation is shown in Fig. 2, demonstrating a concentration/risk relationship during both induction and maintenance therapies. The ROC analyses (Fig. 3) suggest that infliximab concentrations during maintenance therapy (after infusion 3) had the strongest influence on the risk of ADAb formation with an area under the curve (AUC) of 0.76 (CI 0.71–0.80). The serum infliximab cut‐off that best discriminated between patients with and without later ADAb formation was 5 mg/L (sensitivity 71% [CI 55–83] and specificity 74% [CI 69–79]) during maintenance. At infusions 2 and 3, the AUC was 0.66 (CI 0.61–0.70) and 0.76 (CI 0.71–0.80), respectively, and the cut‐offs were 26 mg/L (sensitivity 69% [CI 57–78] and specificity 67% [CI 62–72]) and 19 mg/L (sensitivity 71.0% [CI 59–80] and specificity 76% [CI 71–80]), respectively.

## Discussion

In this study of 410 patients with immune‐mediated inflammatory diseases initiating infliximab therapy, ADAb formation occurred in 19% of patients during the first 38 weeks of infliximab treatment. Thus, a large proportion of patients is at risk of treatment failure and immunogenicity‐related side effects. Several risk factors for ADAb formation were identified: diagnosis of RA, lifetime smoking, infliximab monotherapy, higher disease activity, lower infliximab doses, “drug holidays” of more than 11 weeks, and lower infliximab serum concentrations. This knowledge provides a basis for treatment strategies to mitigate the formation of ADAb and for identifying patients in whom these measures are of particular importance.

An association between low infliximab drug concentrations and subsequent immunogenicity has previously been proposed [13, 16, 23], but the concentration necessary to mitigate ADAb formation is not known. This large prospective study confirmed the relationship between low drug concentrations and immunogenicity and suggested target drug concentrations to reduce the risk of ADAb formation. We identified the drug concentrations to best discriminate patients with and without later ADAb formation to be 26, 19, and 5 mg/L at infusions 2 and 3 and during maintenance therapy (after infusion 3), respectively. The concentration/risk curves suggested that drug concentrations above these levels did not further reduce the risk of ADAb formation. These results imply that slightly higher infliximab concentrations are needed to prevent ADAb formation than to ensure effectiveness [17, 19]. This information needs to be balanced against the increased costs and possible increased risk of infections with higher doses of infliximab [24].

We found a reduced risk of immunogenicity for higher infliximab doses during the maintenance phase. However, this did not apply during the induction phase. As serum drug concentrations were significantly associated with ADAb formation during the induction phase, the lack of association between drug doses and ADAb may indicate that factors other than dosing, such as disease activity, influence variations in drug concentrations to a greater extent during induction than during maintenance treatment with infliximab.

Prior studies have shown a high rate of immunogenicity in patients receiving episodic “on demand” infliximab treatment [25], stressing the importance of ensuring continuous, scheduled infliximab therapy. The risk of immunogenicity posed by single short‐term pauses in therapy such as pauses recommended in relation to surgery and infections, has not been established. Our study demonstrated that the risk of immunogenicity increased more than fourfold in patients with an infusion interval of more than 11 weeks. As drug holidays are unavoidable in some cases, these data underscore the importance of keeping these delays as short as possible, at most 11 weeks.

Consistent with several previous reports, the use of concomitant immunosuppressive medications lowered the risk of immunogenicity in our study [7, 13, 26, 27]. The proposed dose‐dependent protective effects of thiopurines or methotrexate could not be confirmed by our results [13, 28]. This may be due to the high doses of methotrexate used by most patients in this study. The use of methotrexate or sulfasalazine co‐medication in the RA and PsA subgroups was not significantly protective. This finding is most likely explained by the fact that most patients (90% and 80% with RA and PsA, respectively) used concomitant immunosuppressive medication, mainly methotrexate. In the SpA subgroup, none of the 27 patients who used concomitant immunosuppressive medications (25 methotrexate and 2 sulfasalazine) developed ADAb, compared to 13 of 92 patients who did not. This finding suggests that, contrary to current clinical practice, the use of concomitant immunosuppressive medication may be valuable in patients with SpA. This finding is supported by observational data showing increased drug survival of TNFi in patients with SpA on concomitant immunosuppressive medication [29].

High disease activity has been associated with increased immunogenicity [14, 30]. Patients with an activated immune system are probably more likely to develop an immune response to the drug [31], partly due to increased infliximab clearance [13]. In line with this, higher disease activity assessed by mean ESR and CRP levels increased the risk of ADAb formation in our study. The observed association between ADAb and glucocorticoid therapy during follow‐up likely represents patients with flares.

We have shown that lifetime smoking almost doubles the odds of ADAb formation. Similar findings have been reported in studies of patients with CD and RA [13, 14]. Smoking has been shown to alter both humoral and cell‐mediated immune responses [32]; however, the exact mechanism behind this finding remains unknown.

To our knowledge, this is the first study to assess risk factors for immunogenicity across all six diagnoses for which infliximab is approved. Patients with SpA had a significantly lower risk of immunogenicity than those with other diagnoses, while the highest rate of immunogenicity was seen in patients with RA, for whom the risk of ADAb formation was almost doubled compared to that of the other diagnoses. This finding was unexpected, as prior data have indicated high immunogenicity rates in patients with inflammatory bowel diseases [7]. High immunogenicity rates in RA patients, despite the high proportion of patients on concomitant medication compared to SpA patients [33, 34], might be partly explained by the fact that RA patients receive a lower starting dose of infliximab than patients with other diagnoses (3 mg/kg vs. 5 mg/kg). Patients with SpA, however, did not receive higher doses than patients with the other diagnoses but still had the highest serum drug concentrations and the lowest rate of ADAb formation. Although these observational data cannot establish a causal link between the risk factors identified and ADAb formation, our findings indicate that not only treatment‐related factors but also disease‐specific mechanisms influence both drug concentrations and immunogenicity. Cytokines that can activate B cells, including interleukin‐6 and B‐cell activating factor, have been suggested to contribute to the pathogenesis of RA [35, 36, 37] and may also promote immunogenicity [37, 38]. This may explain some of the differences in immunogenicity rates between RA and other diseases, where these cytokines are thought to be less important.

Our study identified risk factors for ADAb formation in patients treated with infliximab, and further research should address whether these risk factors are also of significance in patients treated with less immunogenic TNFi. Prior data, however, suggest some of the same risk factors for ADAb formation to adalimumab which exhibits a similar immunogenic profile as infliximab [13, 14].

The ADAb assay used in this study reports ADAb in the 15–200 µg/L range, which is comparable to the ranges of several commercially available assays. However, different assays for the detection of ADAb are in use, and ADAb concentrations are not always comparable between studies [39]. We used a drug‐sensitive assay with limited ability to detect ADAb in the presence of drug. Although infliximab concentrations measured at the same time point to or later than the detection of ADAb were not included in our regression analyses and concentration/risk relationship assessments, we cannot exclude the possibility that the low drug concentrations measured prior to detection of the first ADAb‐positive sample could have been a result of incipient ADAb formation in some patients. Therefore, a cause–effect relationship cannot be established based on this study. With a drug‐tolerant assay, ADAb formation could potentially have been detected earlier in some patients and provided more insight into the timely development of ADAb in relation to drug concentrations. However, low concentrations of ADAb in the presence of drug are often transient or of minor clinical relevance [31, 40], and studies have indicated a lack of additional clinical value of drug‐tolerant assays [41, 42, 43]. Drug‐tolerant assays usually involve elaborate preanalytical acid dissociation steps, which are rarely feasible in routine practice. Other limitations include the risk of damage/inactivation of ADAb molecules due to low pH. Additionally, we used an assay designed to detect neutralising antibodies only, as these are presumably the most clinically relevant ADAb [44].

One of the strengths of our study is the inclusion of six different diagnoses, which provides new insights into immunogenicity in immune‐mediated inflammatory diseases in general. Further strengths include the study design, the large number of included patients, the high retention rate, and the fact that all serum samples were obtained as trough samples.

This study has some limitations. The large number of univariate analyses performed confers a possible bias. Of the 15 significant risk factors in the univariate analyses, 12 remained significant after controlling for multiple testing. The three variables that did not stand up to multiplicity adjustment in univariate analyses (having more than 11 weeks between infusions, having one or more infliximab dose increments, and age) should therefore be interpreted with caution. Furthermore, this study was not powered to conduct robust subgroup analyses. Therefore, these analyses should be regarded as explorative, and further studies are needed to confirm our results. However, these analyses still serve as an indication of the most influential factors in each subgroup.

In conclusion, this large prospective study of patients with immune‐mediated inflammatory diseases starting infliximab therapy identified several patient‐ and treatment‐related risk factors for immunogenicity and suggests optimal cut‐offs for drug concentrations to prevent ADAb formation. Ensuring an optimal infliximab dose, use of concomitant immunosuppressive medication, and avoiding “drug holidays” longer than 11 weeks are measures that can mitigate the formation of ADAb and thus improve effectiveness of infliximab therapy, particularly for patients with other risk factors for immunogenicity such as smoking or a diagnosis of RA. Proactive TDM to ensure optimal doses and timely identification of ADAb formation might also be useful in at‐risk patients to prevent treatment failure and infusion reactions [17]. For patients exhibiting risk factors where these treatment strategies are not feasible, treatment with a less immunogenic TNFi or other biologic or targeted synthetic drugs might be a better choice.

## Author contributions

Marthe Kirkesæther Brun: Conceptualization; Data curation; Formal analysis; Investigation; Methodology; Project administration; Resources; Software; Validation; Visualization; Writing – original draft; Writing – review & editing. Joseph Sexton: Conceptualization; Data curation; Formal analysis; Investigation; Methodology; Resources; Software; Validation; Visualization; Writing – review & editing. Johanna Elin Gehin: Conceptualization; Investigation; Methodology; Resources; Visualization; Writing – review & editing. Øystein Sandanger: Conceptualization; Investigation; Methodology; Resources; Writing – review & editing. Inge Christoffer Olsen: Conceptualization; Investigation; Methodology; Resources; Visualization; Writing – review & editing. Rolf Anton Klaasen: Investigation; Methodology; Resources; Visualization; Writing – review & editing. Cato Mørk: Conceptualization; Investigation; Methodology; Resources; Writing – review & editing. Tore K. Kvien: Conceptualization; Funding acquisition; Investigation; Methodology; Visualization; Writing – review & editing. Jørgen Jahnsen: Conceptualization; Investigation; Methodology; Resources; Supervision; Writing – review & editing. Nils Bolstad: Conceptualization; Investigation; Methodology; Resources; Supervision; Visualization; Writing – review & editing. Espen A. Haavardsholm: Conceptualization; Funding acquisition; Investigation; Methodology; Resources; Supervision; Visualization; Writing – original draft; Writing – review & editing. Silje Watterdal Syversen: Conceptualization; Funding acquisition; Investigation; Methodology; Project administration; Resources; Supervision; Visualization; Writing – original draft; Writing – review & editing

## Conflicts of interest

Dr. Brun has nothing to disclose. Dr. Goll reports personal fees from AbbVie, personal fees from Pfizer, personal fees from Sandoz, personal fees from Celltrion, personal fees from Lilly, personal fees from UCB, and personal fees from Boehringer Ingelheim outside the submitted work. Dr. Jørgensen reports personal fees from BMS, personal fees from Roche, and Celltrion grants from South‐Eastern Norway Regional Health Authorities. Dr. Sexton has nothing to disclose. Dr. Gehin has nothing to disclose. Dr. Sandanger has nothing to disclose. Dr. Olsen has nothing to disclose. Dr. Klaasen has nothing to disclose. Dr. Warren has nothing to disclose.

Dr. Mørk reports personal fees from Novartis Norge AS, personal fees from LEO Pharma AS, personal fees from ACO Hud Norge AS, personal fees from Cellgene AS, personal fees from Abbvie, and personal fees from Galderma Nordic AB and Eli‐Lilly outside the submitted work.

Dr. Kvien reports grants from the Norwegian Regional Health Authorities (inter‐regional KLINBEFOSK grants) and grants from South‐Eastern Norway Regional Health Authorities during the conduct of the study, grants from AbbVie, grants from Amgen, personal fees from Amgen, grants from BMS, personal fees from Celltrion, personal fees from Egis, personal fees from Evapharma, personal fees from Ewopharma, personal fees from Gilead, personal fees from Hikma, personal fees from Mylan, grants from Novartis, personal fees from Oktal, grants from Pfizer, personal fees from Pfizer, personal fees from Sandoz, personal fees from Sanofi, grants from UCB, and personal fees from UCB outside the submitted work. Dr. Jahnsen has nothing to disclose. Dr. Bolstad reports personal fees from Roche, personal fees from Janssen, and personal fees from Novartis outside the submitted work. Dr. Haavardsholm reports grants from Norwegian Regional Health Authorities (inter‐regional KLINBEFORSK grants) and grants from the South‐Eastern Norway Regional Health Authority during the conduct of the study, personal fees from Pfizer, personal fees from AbbVie, personal fees from Celgene, personal fees from Novartis, personal fees from Janssen, personal fees from Gilead, personal fees from Eli‐Lilly, and personal fees from UCB outside the submitted work. Dr. Syversen reports grants from Norwegian Regional Health Authorities and grants from South‐Eastern Norway Regional Health Authorities during the conduct of the study and personal fees from Thermo Fisher outside the submitted work.

## Supporting information


**Appendix Table 1**. Treatment algorithm in the therapeutic drug monitoring arm
**Appendix Table 2**. Description of the ADAb assay
**Appendix Table 3**. Infliximab drug doses and serum drug concentrations in the maintenance phase
**Appendix Table 4**. Transient anti‐drug antibody formation
**Appendix Table 5a‐b**. Sensitivity analyses of risk factors for anti‐drug antibody formation
**Appendix Table 6**. Multiplicity adjustment
**Appendix Table 7a‐c**. Risk factors in disease subgroups
