# Supplementary material for: Risk factors for anti‐drug antibody formation to infliximab: Secondary analyses of a randomised controlled trial
Source: J Intern Med. 2022 Apr 26;292(3):477–91. doi: 10.1111/joim.13495 (PMC9545769; doi:10.1111/joim.13495)
Supplement: Supplementary file 1 — Appendix Table 1. Treatment algorithm in the therapeutic drug monitoring arm Appendix Table 2. Description of the ADAb assay Appendix Table 3. Infliximab drug doses and serum drug concentrations in the maintenance phase Appendix Table 4. Transient anti‐drug antibody formation Appendix Table 5a‐b. Sensitivity analyses of risk factors for anti‐drug antibody formation Appendix Table 6. Multiplicity adjustment Appendix Table 7a‐c. Risk factors in disease subgroups [file JOIM-292-477-s001.docx]

**Supplementary appendix**

Brun MK, Goll GL, Jørgensen KK, et al. Risk factors for anti-drug antibody formation to infliximab: secondary analyses of a randomized controlled trial

**Table of contents**

[Appendix Table 1. Treatment algorithm in the therapeutic drug monitoring arm 2](#_Toc92965139)

[Appendix Table 2. Description of the ADAb assay 3](#_Toc92965140)

[Appendix Table 3. Infliximab drug doses and serum drug concentrations in the maintenance phase 4](#_Toc92965141)

[Appendix Table 4. Transient anti-drug antibody formation 5](#_Toc92965142)

[Appendix Table 5a-b. Sensitivity analyses of risk factors for anti-drug antibody formation 6](#_Toc92965143)

[Appendix Table 6. Multiplicity adjustment 8](#_Toc92965144)

[Appendix Table 7a-c. Risk factors in disease subgroups 10](#_Toc92965145)

# Appendix Table 1. Treatment algorithm in the therapeutic drug monitoring arm

| **Infusion number 1-4** | | | **Infusion 5 and onwards** | | |
| --- | --- | --- | --- | --- | --- |
| **Serum infliximab** | Increase dose if:  **<20.0 mg/L** at infusion #2  **<15.0 mg/L** at infusion #3  **<3.0 mg/L** at infusion #4  Increase dose if:  **ADAb^a^ ≤50 µg/L**    Switch therapy if:  **ADAb^a^ >50 µg/L** | No action  (Within target range)  **≥20.0 mg/L** at infusion 2  **≥15.0 mg/L** at infusion 3  **≥3.0 mg/L** at infusion 4 | Increase dose if:  **≤2.0 mg/L**  Consider increasing dose if  **2.1 – 2.9 mg/L**  Increase dose if  **ADAb^a^ ≤50 µg/L**    Switch therapy if  **ADAb^a^ >50 µg/L** | No action  (Within target range)  **3.0 – 8.0 mg/L** | Consider decreasing dose if  **8.1 – 10.0 mg/L**  Decrease dose if  **>10.0 mg/L** |
| **Guideline for action:**  Infusion 1-4: Increase the dose by 2 weeks shortening of the infusion interval.  Infusion 5 and onwards: Increase the dose by 2-2,5 mg/kg increase in given dose (maximum dose of 10 mg/kg) or by 2 weeks shortening of the infusion interval (minimum interval of 4 weeks). Decrease the dose by 2-2,5 mg/kg decrease in given dose or by 2 weeks increase in infusion interval (maximum interval of 10 weeks).  ^a^ADAb=Anti-drug antibodies | | | | | |

# Appendix Table 2. Description of the ADAb assay

| 1 | Patient sample, europium-labelled infliximab F(ab’)_2_ and assay buffer are added to streptavidin-coated assay wells. ADAb in patient sample will bind to infliximab. (60-minute incubation.) |
| --- | --- |
| 2 | Biotinylated TNF is added to streptavidin-coated assay wells, and immobilized through the strong interaction between biotin and streptavidin. Europium-labelled infliximab F(ab’)2 not neutralized by ADAb will bind to TNF. (60-minute incubation.) |
| 3 | Unbound europium-labelled infliximab F(ab’)_2_ is removed by wash. Enhancement solution is added to assay wells (10-minute incubation). Fluorescence from europium is counted by the instrument, signal intensity is inversely proportional to amount of ADAb in sample. Measuring range 15-200 µg/L. |
| The ADAb assay uses the affinity matured HCA233 monoclonal antibody, which has a very high affinity (KD=0.12 nM) as a calibrator antibody. The assay is fully automated on the AutoDELFIA immunoassay platform. ADAb = Anti-drug antibodies; F(ab’)2 = Fragment antigen binding; TNF = Tumour necrosis factor. | |

# Appendix Table 3. Infliximab drug doses and serum drug concentrations in the maintenance phase

| **Median weekly drug doses in the maintenance phase* (IQR), mg/kg/week** |  |
| --- | --- |
| Total (n=355†) | 0.8 (0.7-1.0) |
| Rheumatoid arthritis (n=71) | 0.5 (0.5-0.7) |
| Psoriatic arthritis (n=36) | 0.9 (0.8-1.0) |
| Spondyloarthritis (n=97) | 0.8 (0.7-0.9) |
| Ulcerative colitis (n=77) | 0.9 (0.8-1.3) |
| Crohn’s disease (n=53) | 0.9 (0.9-1.0) |
| Psoriasis (n=21) | 0.8 (0.6-0.9) |
| **Median infliximab concentration during the maintenance phase‡§ (IQR), mg/L** |  |
| Total (n=326\|\|) | 7.0 (4.1-11.3) |
| Rheumatoid arthritis (n=55) | 4.7 (2.3-6.8) |
| Psoriatic arthritis (n=35) | 6.4 (3.8-10.0) |
| Spondyloarthritis (n=95) | 8.3 (5.4-12.8) |
| Ulcerative colitis (n=72) | 9.1 (5.6-13.0) |
| Crohn’s disease (n=50) | 6.6 (4.6-11.0) |
| Psoriasis (n=19) | 5.4 (3.2-7.8) |
| Data are median (IQR). *Infusion 3 and onwards. †Some patients did not have 3 or more infusions. ‡After infusion 3. §Calculated mean for each patient. \|\|Some patients did not have infliximab serum level assessments after infusion 3. | |

# Appendix Table 4. Transient anti-drug antibody formation

| **Patient** | **Diagnosis** | **Infusion number* where ADAb was detected** | **ADAb concentration (µg/L)** |
| --- | --- | --- | --- |
| 1 | Spondyloarthritis | 4 | 16 |
| 2 | Rheumatoid arthritis | 4 | 35 |
| 3 | Rheumatoid arthritis | 6-7 | 26-33 |
| 4 | Rheumatoid arthritis | 4 | 19 |
| 5 | Ulcerative colitis | 3 and 6 | 35 and 17 |
| 6 | Ulcerative colitis | 4 | 89 |
| 7 | Crohn’s disease | 5 | 18 |
| 8 | Psoriasis | 4 | 29 |
| 9 | Ulcerative colitis | 8-9 | 20-32 |
| *Time point for blood serum sampling is trough, 0-7 days before the denoted infusion number.   ADAb = Anti-drug antibodies | | | |

# Appendix Table 5a-b. Sensitivity analyses of risk factors for anti-drug antibody formation

**Appendix Table 5a.** Sensitivity analyses of baseline risk factors for anti-drug antibody formation

| **Anti-drug antibody formation, n= 78/410** | | | | |
| --- | --- | --- | --- | --- |
|  | **Adjusted for age, sex  and diagnosis** | | **Adjusted for age, sex,  diagnosis and CRP** | |
|  | **OR [95% CI]** | ***P*-value** | **OR [95% CI]** | ***P*-value** |
| Age | 1.01 [1.00,1.03] | 0.16 | 1.01 [1.00,1.03] | 0.15 |
| Diagnosis of rheumatoid arthritis (n=84/410) | 1.93 [1.04,3.59] | 0.04 | 1.96 [1.06,3.63] | 0.03 |
| Diagnosis of spondyloarthritis (n=119/410) | 0.44 [0.23,0.85] | 0.01 | 0.44 [0.23,0.84] | 0.01 |
| Prior use of ≥1 TNF inhibitor(s) (n=94/410) | 0.62 [0.31,1.21] | 0.16 | 0.61 [0.31,1.20] | 0.16 |
| Concomitant immunosuppressive therapy* (n=226/410) | 0.40 [0.21,0.76] | <0.01 | 0.40 [0.21,0.76] | <0.01 |
| Concomitant use of prednisolone ≥15mg/day (n=30/410) | 0.26 [0.06,1.14] | 0.07 | 0.26 [0.06,1.13] | 0.07 |
| Infliximab starting dose (mg/kg) (n=410) | 0.83 [0.60,1.16] | 0.28 | 0.83 [0.60,1.16] | 0.28 |
| Lifetime smoking (n=233/410) | 2.00 [1.12,3.58] | 0.02 | 2.00 [1.12,3.59] | 0.02 |
| Results are presented as odds ratios (OR) with 95% confidence intervals (CI). All baseline variables with a *P*-value <0.25 in univariate analysis (Table 2) were included in the sensitivity analyses except for Patient's global assessment of disease activity because this variable was not adjusted for disease activity.  *Included in table despite an unadjusted *P*-value>0.25 because this variable was significant in adjusted analysis. | | | | |

**Appendix Table 5b.** Sensitivity analyses of patient and treatment related risk factors for anti-drug antibody formation during follow-up.

| **Anti-drug antibody formation, n= 77/406*** | | | | |
| --- | --- | --- | --- | --- |
|  | **Adjusted for age, sex  and diagnosis** | | **Adjusted for age, sex,  diagnosis and CRP** | |
|  | **OR [95% CI]** | ***P*-value** | **OR [95% CI]** | ***P*-value** |
| Concomitant use of systemic glucocorticoids after baseline (n=58/406) | 5.83 [3.07,11.08] | <0.001 | 5.70 [2.98,10.93] | <0.001 |
| Infliximab dose/week infusion 1 and 2 (mg/kg/week) (n=390†) | 0.61 [0.27,1.35] | 0.22 | 0.56 [0.25,1.26] | 0.16 |
| Infliximab dose/week infusion 3 and onwards (mg/kg/week) (n=355‡) | 0.14 [0.04,0.49] | <0.01 | 0.14 [0.04,0.48] | <0.01) |
| One or more infliximab dose increment(s) (n=148/406) | 0.46 [0.26,0.83] | 0.01 | 0.46 [0.25,0.83] | 0.01 |
| More than 11 weeks between infusions (n=13/406) | 3.97 [1.21,13.03] | 0.02 | 3.66 [1.12,11.98] | 0.03 |
| Infliximab concentration before infusion 2 (n=398§) | 0.95 [0.93,0.98] | <0.01) | 0.96 [0.93,0.99] | 0.01 |
| Infliximab concentration before infusion 3 (n=389\|\|) | 0.91 [0.88,0.94] | <0.001 | 0.92 [0.89,0.94] | <0.001 |
| Mean infliximab concentration after infusion 3 (n=326¶) | 0.72 [0.63,0.83] | <0.001 | 0.73 [0.64,0.83] | <0.001 |
| Results are presented as odds ratios (OR) with 95% confidence intervals (CI). All follow-up variables with a *P*-value <0.25 in univariate analysis (Table 3) were included in the sensitivity analyses except for mean CRP and mean ESR because these variables were not adjusted for disease activity. CRP = C-reactive protein; ESR = Erythrocyte sedimentation rate. *4 patients did not have assessments after baseline. †Some patients did not have more than one infusion. ‡Some patients did not have 3 or more infusions. §Some patients did not have more than one infusion or did not have a serum infliximab assessment before infusion 2. \|\|Some patients did not have more than two infusions or did not have a serum infliximab assessment before infusion 3. ¶ Some patients did not have more than three infusions or did not have serum infliximab assessments after infusion 3. | | | | |

# Appendix Table 6. Multiplicity adjustment

| **Variable #** | **Variable** | ***P*-value  (unadjusted)** | **Significant (unadjusted)** | **FDR (0.05)** |
| --- | --- | --- | --- | --- |
| 1 | Infliximab concentration  before infusion 3 | <0.0001 | 1 | 1 |
| 2 | Concomitant use of  systemic glucocorticoids  after baseline | <0.0001 | 1 | 1 |
| 3 | Mean infliximab  concentration after  infusion 3 | <0.0001 | 1 | 1 |
| 4 | Mean ESR level | <0.0001 | 1 | 1 |
| 5 | Infliximab concentration  before infusion 2 | 0.0001 | 1 | 1 |
| 6 | Infliximab dose/week  infusion 3 and onwards | 0.0001 | 1 | 1 |
| 7 | Smoking | 0.0038 | 1 | 1 |
| 8 | Diagnosis of  rheumatoid arthritis | 0.0056 | 1 | 1 |
| 9 | Infliximab dose/week  infusion 1 and 2 | 0.0072 | 1 | 1 |
| 10 | Mean CRP level | 0.0080 | 1 | 1 |
| 11 | Diagnosis of  spondyloarthritis | 0.0089 | 1 | 1 |
| 12 | Infliximab starting dose | 0.0101 | 1 | 1 |
| 13 | More than 11 weeks  between infusions | 0.0175 | 1 | 0 |
| 14 | One or more infliximab  dose increment(s) | 0.0184 | 1 | 0 |
| 15 | Age | 0.0254 | 1 | 0 |
| 16 | Patient's global  assessment of disease  activity | 0.0625 | 0 | 0 |
| ... | ... | ... | ... | ... |
| 37 | Disease duration | 0.8591 | 0 | 0 |
| Univariate risk factor associations with ADAb formation. Column «Significant (unadjusted)» shows which variables are significant (=1 if yes, 0 otherwise) as 5% level, but not adjusting for multiplicity. Multiplicity adjusted significance for False discovery rate (5% FDR) is given in column «FDR (0.05)». FDR = false discovery rate; ESR = erythrocyte sedimentation rate; CRP = C-reactive protein; ADAb = anti-drug antibody. | | | | |

# Appendix Table 7a-c. Risk factors in disease subgroups

**Appendix Table 7a.** Risk factors for antidrug antibody formation in the peripheral arthritis (rheumatoid arthritis and psoriatic arthritis) subgroup

|  | **Anti-drug antibody formation, n=36/128** | | | |
| --- | --- | --- | --- | --- |
|  | **Univariate analysis** | | **Adjusted analysis** | |
|  | **OR [95% CI]** | ***P*-value** | **OR [95% CI]** | ***P*-value** |
| **Baseline variables*** |  |  |  |  |
| Lifetime smoking | 2.79 [1.11,7.03] | 0.02 | 3.02 [1.08,8.39] | 0.02 |
| Randomized to TDM | 0.51 [0.23,1.12] | 0.03 | 0.50 [0.22,1.13] | 0.03 |
| **Follow-up variables†** |  |  |  |  |
| Concomitant use of systemic glucocorticoids after baseline | 3.50 [1.29,9.55] | 0.01 | 3.39 [1.20,9.54] | 0.02 |
| One or more infliximab dose increment(s) | 0.51 [0.22,1.22] | 0.13 | 0.45 [0.18,1.11] | 0.08 |
| More than 11 weeks between infusions | 5.56 [0.97,31.84] | 0.05 | 5.75 [0.92,35.74 | 0.06 |
| Mean DAS28 level | 1.46 [1.01,2.09] | 0.04 | 1.54 [1.05,2.25] | 0.03 |
| Mean CRP level | 1.09 [1.01,1.17] | 0.03 | 1.08 [1.01,1.17] | 0.03 |
| Mean ESR level | 1.05 [1.01,1.09] | <0.01 | 1.06 [1.02,1.10] | <0.01 |
| Mean CDAI level | 1.03 [0.98,1.08] | 0.22 | 1.03 [0.98,1.09] | 0.19 |
| Mean SDAI level | 1.03 [0.99,1.08] | 0.18 | 1.04 [0.99,1.09] | 0.15 |
| Infliximab level at infusion 2 | 0.97 [0.93,1.01] | 0.13 | 0.96 [0.92,1.01] | 0.09 |
| Infliximab level at infusion 3 | 0.94 [0.90,0.98] | <0.01 | 0.94 [0.89,0.98] | <0.01 |
| Mean infliximab level after infusion 3 | 0.74 [0.59,0.93] | 0.01 | 0.72 [0.56,0.94] | 0.02 |
| Results are presented as odds ratios (OR) with 95% confidence intervals (CI). The adjusted analyses are corrected for age, gender and disease activity (DAS28). All variables with a *P*-value <0.25 in univariate analysis included. Variables tested, but not associated with ADAb (P>0.25 in univariate analysis) includes *at baseline: gender, BMI, disease duration, prior TNFi and other biologic therapy, disease activity parameters, concomitant use of methotrexate, sulfasalazine or systemic glucocorticoids and infliximab starting dose. †after infliximab treatment initiation: weekly drug doses and having one or more infections. TDM, therapeutic drug monitoring; DAS28, disease activity score in 28 joints; CRP, C-reactive protein; ESR, Erythrocyte sedimentation rate; CDAI, simplified disease activity index; SDAI simplified disease activity index. | | | | |

**Appendix Table 7b.** Risk factors for antidrug antibody formation in the spondyloarthritis subgroup

|  | **Anti-drug antibody formation, n=13/119** | | | | | |
| --- | --- | --- | --- | --- | --- | --- |
|  | **Univariate analysis** | | | **Multivariate analysis** | | |
|  | **OR [95% CI]** | | ***P*-value** | **OR [95% CI]** | | ***P*-value** |
| **Baseline variables*** |  |  |  |  |  |  |
| Age, years | 0.97 | [0.93,1.02] | 0.21 | 0.97 | [0.93,1.02] | 0.21 |
| HLA-B27 positive | 0.36 | [0.10,1.25] | 0.11 | 0.28 | [0.07,1.08] | 0.07 |
| Concomitant immunosuppressive therapy‡ | - | - | - | - | - | - |
| Concomitant use of systemic glucocorticoids‡ | - | - | - | - | - | - |
| **Follow-up variables†** |  |  |  |  |  |  |
| Concomitant use of systemic glucocorticoids after baseline | 4.27 | [1.11,16.39] | 0.03 | 3.96 | [0.98,16.02] | 0.05 |
| Mean ASDAS level | 1.79 | [0.78,4.15] | 0.17 | 2.14 | [0.89,5.17] | 0.09 |
| Mean CRP level | 1.05 | [1.01,1.08] | <0.01 | 1.05 | [1.01,1.08] | <0.01 |
| Mean ESR level | 1.05 | [1.03,1.07] | <0.001 | 1.05 | [1.02,1.07] | <0.001 |
| Infliximab level at infusion 2 | 0.92 | [0.86,0.99] | 0.02 | 0.92 | [0.85,0.99] | 0.02 |
| Infliximab level at infusion 3 | 0.80 | [0.71,0.90] | <0.001 | 0.79 | [0.70,0.90] | <0.001 |
| Mean infliximab level after infusion 3 | 0.61 | [0.44,0.85] | <0.01 | 0.63 | [0.44,0.91] | 0.01 |
| Results are presented as odds ratios (OR) with 95% confidence intervals (CI). The adjusted analyses are corrected for age, gender and disease activity (ASDAS). All variables with a *P*-value <0.25 in univariate analysis included. Variables tested, but not associated with ADAb (*P*-values >0.25 in univariate analysis) includes *at baseline: gender, BMI, disease duration, prior TNFi and other biologic therapy, disease activity parameters, and infliximab starting dose. †After infliximab treatment initiation: Weekly drug doses, having one or more infliximab dose increments and having more than 11 weeks between infusions. ‡None of the patients usig concomitant methotrexate, sulfasalazine or systemic glucocorticoids at baseline had ADAb formation. HLA, Human leucocyte antigen; ASDAS Ankylosing Spondylitis Disease Activity Score; CRP, C-reactive protein; ESR, Erythrocyte sedimentation rate. | | | | | | |

**Appendix Table 7c.** Risk factors for antidrug antibody formation in the inflammatory bowel disease (Ulcerative colitis and Crohn’s disease) subgroup

|  | **Anti-drug antibody formation, n=24/141** | | | | | |
| --- | --- | --- | --- | --- | --- | --- |
|  | **Univariate analysis** | | | **Multivariate analysis** | | |
|  | **OR [95% CI]** | | ***P*-value** | **OR [95% CI]** | | ***P*-value** |
| **Baseline variables*** |  |  |  |  |  |  |
| Age, years | 1.04 | [1.01,1.07] | 0.01 | 1.04 | [1.01,1.07] | 0.01 |
| Concomitant use of thiopurines at baseline | 0.26 | [0.08,0.80] | 0.02 | 0.29 | [0.09,0.93] | 0.04 |
| Hemoglobin level (g/100ml) | 0.80 | [0.62,1.03] | 0.08 | 0.84 | [0.63,1.11] | 0.22 |
| **Follow-up variables†** |  |  |  |  |  |  |
| Concomitant use of systemic glucocorticoids after baseline | 9.36 | [3.40,25.79] | <0.001 | 9.47 | [3.17,28.23] | <0.001 |
| Infliximab dose/week infusion 3 and onwards (mg/kg/week) | 0.07 | [0.01,0.44] | <0.01 | 0.05 | [0.01,0.35] | <0.01 |
| One or more infliximab dose increment(s) | 0.32 | [0.11,0.93] | 0.04 | 0.30 | [0.10,0.90] | 0.03 |
| More than 11 weeks between infusions§ | - | - | - | - | - | - |
| Mean HBI level‡ | 1.23 | [0.99,1.51] | 0.06 | 1.22 | [0.98,1.53] | 0.08 |
| Mean CRP level | 1.05 | [1.00,1.10] | 0.05 | 1.04 | [0.99,1.10] | 0.09 |
| Mean ESR level | 1.07 | [1.02,1.12] | <0.01 | 1.06 | [1.01,1.11] | 0.02 |
| Mean albumin level (U/L) | 0.90 | [0.80,1.02] | 0.09 | 0.96 | [0.84,1.10] | 0.6 |
| Mean white blood cell count (10^9^/L) | 1.33 | [1.04,1.72] | 0.03 | 1.34 | [1.03,1.75] | 0.03 |
| Infliximab level at infusion 2 | 0.97 | [0.93,1.01] | 0.1 | 0.97 | [0.93,1.02] | 0.2 |
| Infliximab level at infusion 3 | 0.93 | [0.88,0.98] | <0.01 | 0.94 | [0.89,0.99] | 0.01 |
| Mean infliximab level after infusion 3 | 0.84 | [0.70,0.99] | 0.04 | 0.84 | [0.71,1.00] | 0.05 |
| Having one or more infections | 0.30 | [0.07,1.34] | 0.11 | 0.24 | [0.05,1.16] | 0.08 |
| Results are presented as odds ratios (OR) with 95% confidence intervals (CI). The adjusted analyses are corrected for age, gender and disease activity (standardized disease activity). All variables with a *P*-value <0.25 in univariate analysis included. Variables tested, but not associated with ADAb (*P*-values >0.25 in univariate analysis) includes *at baseline: gender, BMI, disease duration, prior TNFi and other biologic therapy, concomitant use of glucocorticoids, white blood cell count and albumin levels, disease activity parameters including calprotectin levels, and infliximab starting dose. †After infliximab treatment initiation: Mean hemoglobin, calprotectin and PMS (ulcerative colitis only) levels. ‡Only Crohn’s disease (n=58). §The one patient with an infusion interval of more than 11 weeks had ADAb formation. ESR, Erythrocyte sedimentation rate; CRP, C-reactive protein; HBI, Harvey Bradshaw Index; PMS, Partial Mayo Score. | | | | | | |
